# Supplementary material for: Knowledge, attitudes and preventive practices towards COVID-19 among workers at two points of entry in South-Eastern Botswana: A cross-sectional study
Source: PLoS One. 2022 Nov 10;17(11):e0277506. doi: 10.1371/journal.pone.0277506 (PMC9648788; doi:10.1371/journal.pone.0277506)
Supplement: S1 File — (DOCX) [file pone.0277506.s002.docx]

**COVID-19 Knowledge, Attitudes, Practices and Mental Health Outcomes among Point of Entry staff in Botswana**

**DATA COLLECTION TOOL**

**A. Sociodemographic**

**ID: ________________________ Residence (City/town/village): ____________________________**

**District: _____________________ Border post: ______________________________**

- 1. **Gender**: 1. Male 2. Female
  2. **Age in years:** _____________________
  3. **Nationality**: 1. Botswana 2. Other, Specify: ______________________
  4. **Education level**: 1. No formal education 2. Primary 3 Secondary 4. Diploma 5. Degree or higher
  5. **Marital status**: 1. Married 2. Single 3. Divorced 4. Widowed 5. Other
  6. **Religion**: 1. Christian 2. Muslim 3. Other, specify________________
  7. **Work**: 1. Port health 2. BURS 3. Immigration 4. Police 5. Military 6. Veterinary 7. Other, _______________
  8. **Length of Employment (years):** a. 0-5 b. 6-10 c. 11-15 d. 16-20 e. more than 20
  9. **Monthly Income (BWP):** a. less than 2000 b. 2000-5000 c. 5000-10000 d. more than 10000

**B. Medical History**

1. Have you had flu-like symptoms in the last month? **A. Yes** **B. No**
2. Do you have any chronic medical conditions? **A. Yes B. No**
3. If YES to (2) above, what medical conditions do you have? _______________

4. Are you on any long-term medications? **A. Yes B. No**

5. Do you have any psychiatric illnesses? **A. Yes B. No**

6. Do you have history of depression? **A. Yes B. No**

7. Do you have history of anxiety disorder? **A. Yes B. No**

8. Do you have any of the following conditions?

A. Asthma **Yes No**

B. Heart disease **Yes No**

C. Cancer **Yes No**

9. Have you ever tested for COVID-19? **A. Yes B. No**

10. Have you ever tested positive for COVID-19? **A. Yes B. No**

11. If yes to 10 have you had any chronic/ long term effects/complications of COVID19 infection? **A. Yes B. No**

12. Have you been in quarantine? **A. Yes B. No**

**D. Social History**

1. Do you smoke? **A. Yes B. No**

2. Do you drink alcohol? **A. Yes B. No**

3. Have you experienced stigma/discrimination due to your COVID-19 risk? **A. Yes B. No**

4. Has any of your family members or close friends been isolated or quarantined for COVID-19? **A. Yes B. No**

5. Do you have any of the following groups in your household?

A. Persons over the age of 65 **A. Yes B. No**

B. People with Chronic Lung OR heart disease **A. Yes B. No**

C. People with cancer **A. Yes B. No**

**Table 1 Knowledge and Attitudes**

|  | **Yes** | **No** | **I don’t know** |
| --- | --- | --- | --- |
| **K1.The main clinical symptoms of COVID-19 are fever, dry cough, and muscle pains.** |  |  |  |
| **K2 Young people are more likely to develop severe disease than older people** |  |  |  |
| **K3 Currently there is no cure for COVID-19** |  |  |  |
| **K4 Most people with COVID-19 die** |  |  |  |
| **K5 Most people with COVID-19 develop severe disease** |  |  |  |
| **K6 People with underlying chronic illness are more likely to develop severe disease than the general population** |  |  |  |
| **K7 Persons with COVID-19 cannot transmit the virus to others when they do not have a fever** |  |  |  |
| **K8 The COVID-19 virus spreads via respiratory droplets of infected individuals** |  |  |  |
| **K9 Face masks are effective in reducing the spread of COVID-19** |  |  |  |
| **K10 It is not necessary for children and young adults to take measures to prevent the infection by the COVID-19 virus** |  |  |  |
| **K11 To prevent the infection by COVID-19, individuals should avoid going to crowded places** |  |  |  |
| **K12 Isolation of people who are infected with the COVID-19 virus is an effective way of reducing the spread of the virus.** |  |  |  |
| **K13 People who have contact with someone infected with the COVID-19 virus should be immediately isolated in a proper place.** |  |  |  |
| **A1. Do you agree that COVID-19 will finally be successfully controlled?** |  |  |  |
| **A2. Do you have confidence that Botswana can win the battle against the COVID-19 virus?** |  |  |  |
| **A3. Are you generally happy with Botswana’s response to COVID-19?** |  |  |  |
| **A4. Do you think you are at a significantly increased risk of COVID-19 infection due to the nature of your job?** |  |  |  |
| **A5. Do you believe COVID 19 burden is exaggerated to create fear and/or to restrict people’s freedoms?** |  |  |  |

**Table 2 Practices**

|  | **Never (0)** | **Rarely (1)** | **Sometimes (2)** | **Most of the time (3)** | **Always (4)** |
| --- | --- | --- | --- | --- | --- |
| **P1.In the past 2 weeks, how often have you worn a mask when going out?** |  |  |  |  |  |
| **P2. In the past 2 weeks, how often do you manage to keep a distance of at least 1metre from other people in public places?** |  |  |  |  |  |
| **P3. In the past 2 weeks, how often have you washed your hands or used a sanitizer after interaction with other people or touching shared objects?** |  |  |  |  |  |
| **P4. In the past 2 weeks, how often have you worn a mask correctly (covering nose and mouth) when interacting with colleagues at work?** |  |  |  |  |  |
| **P5. In the past 2 weeks, how often have you NOT managed to keep a distance of at least 1 metre between yourself and colleagues in the work place** |  |  |  |  |  |
| **P6. In the past 2 weeks, how often have you been able to sanitize shared surfaces and objects at least 3 times daily at work?** |  |  |  |  |  |
| **P7. In the past 2 weeks, how often have you had your temperature checked before reporting for work and interacting with your colleagues?** |  |  |  |  |  |
| **P8. In the past 4 weeks, how often have you come to work when you have had flu like symptoms (cough, fever, runny nose, etc)** |  |  |  |  |  |

**Table 3. Patient Health Questionnaire (PHQ)**

| **Over the last 2 weeks how often have you been bothered by any of the following problems?** | **Not at all** | **Several days** | **More than half the days** | **Nearly every day** |
| --- | --- | --- | --- | --- |
| **1.Little interest or pleasure in doing things** |  |  |  |  |
| **2. Feeling down, depressed or hopeless** |  |  |  |  |
| **3.Trouble falling or staying asleep, or sleeping too much** |  |  |  |  |
| **4.Feeling tired or having little energy** |  |  |  |  |
| **5. Poor appetite or overeating** |  |  |  |  |
| **6. Feeling bad about yourself- or that you are a failure or have let yourself or your family down** |  |  |  |  |
| **7. Trouble concentrating on things, such as reading the newspaper or watching television** |  |  |  |  |
| **8. Moving or speaking so slowly that other people could have**  **noticed? Or the opposite being so fidgety and restless that you have been moving around a lot more than usual** |  |  |  |  |
| **9.Thoughts that you would be better off dead or hurting yourself in some way** |  |  |  |  |

**Table 4**

| **Over the last 2 weeks how often have you been bothered by any of the following problems?** | **Not at all** | **Several days** | **More than half the days** | **Nearly every day** |
| --- | --- | --- | --- | --- |
| **1.Feeling nervous, anxious, or on edge** |  |  |  |  |
| **2.Not being able to stop or control worrying** |  |  |  |  |
| **3.Worrying too much about different things** |  |  |  |  |
| **4.Trouble relaxing** |  |  |  |  |
| **5. Being so restless that it’s hard to sit still** |  |  |  |  |
| **6.Becoming easily annoyed or irritable** |  |  |  |  |
| **7.Feeling afraid as if something awful might happen** |  |  |  |  |
