# Supplementary material for: Knowledge, attitudes and preventive practices towards COVID-19 among workers at two points of entry in South-Eastern Botswana: A cross-sectional study
Source: PLoS One. 2022 Nov 10;17(11):e0277506. doi: 10.1371/journal.pone.0277506 (PMC9648788; doi:10.1371/journal.pone.0277506)
Supplement: Attachment — Submitted filename: Response to reviewers161022.docx [file pone.0277506.s004.docx]

**Response to reviewers**

Reviewer #3: The rationale for using 50% or 0.5, as P in the sample size estimation is not very clear. The authors may need to consider using P from a previous study. The assumption that the proportion of individuals with good KAPs would fall between 10% and 90% seems not well substantiated in the light of the fact that knowledge, attitudes and practice were assessed separately.

Response

Thank you for this comment. We have now used a previous similar study in Nigeria where 99.5% and 79.5% of respondents had good knowledge and attitudes respectively. We used the 79.5% to calculate the minimum sample size which comes down to 246 participants. We agree that since knowledge, attitudes and practices were assessed separately, it would be difficult to predict that the KAP would fall between 10% and 90%. We have therefore removed that statement.
